# Best Practices for Designing and Testing Behavioral and Health Communication Interventions for Delivery in Private Facebook Groups: Tutorial

**DOI:** 10.2196/58627

**Published:** 2024-09-04

**Authors:** Sherry Pagoto, Natalie Lueders, Lindsay Palmer, Christie Idiong, Richard Bannor, Ran Xu, Spencer Ingels

**Affiliations:** 1 Department of Allied Health Sciences University of Connecticut Storrs, CT United States; 2 Bold Bear Consulting LLC Cota de Caza, CA United States; 3 University of Massachusetts Chan Medical School Worcester, MA United States; 4 Meta San Francisco, CA United States

**Keywords:** social media, Facebook, behavioral intervention, health communication, Facebook groups

## Abstract

Facebook, the most popular social media platform in the United States, is used by 239 million US adults, which represents 71% of the population. Not only do most US adults use Facebook but they also spend an average of 40 minutes per day on the platform. Due to Facebook’s reach and ease of use, it is increasingly being used as a modality for delivering behavioral and health communication interventions. Typically, a Facebook-delivered intervention involves creating a private group to deliver intervention content for participants to engage with asynchronously. In many interventions, a counselor is present to facilitate discussions and provide feedback and support. Studies of Facebook-delivered interventions have been conducted on a variety of topics, and they vary widely in terms of the intervention content used in the group, use of human counselors, group size, engagement, and other characteristics. In addition, results vary widely and may depend on how well the intervention was executed and the degree to which it elicited engagement among participants. Best practices for designing and delivering behavioral intervention content for asynchronous delivery in Facebook groups are lacking, as are best practices for engaging participants via this modality. In this tutorial, we propose best practices for the use of private Facebook groups for delivery and testing the efficacy of behavioral or health communication interventions, including converting traditional intervention content into Facebook posts; creating protocols for onboarding, counseling, engagement, and data management; designing and branding intervention content; and using engagement data to optimize engagement and outcomes.

## Introduction

The vast majority of the US population, both adults and youth, spends a substantial amount of time online, with social media platforms accounting for a major portion of this time [1]. For example, Facebook (Meta), the most popular social media platform in the United States, is used by 239 million US adults, which represents 71% of the population [2]. In 2023, Facebook users spent on average 30 minutes per day on the platform [1]. In a survey of US adults about the top 3 mobile apps they felt they could least do without, Facebook was the most frequently cited app [3]. To increase the richness of users’ experiences on Facebook, in 2010, Facebook launched groups, which are private spaces for people to come together around specific topics of interest (eg, hobbies and health). In 2017, Facebook founder, Mark Zuckerberg, announced an increased investment in Facebook groups, explaining, “building a global community that works for everyone starts with the millions of smaller communities and intimate social structures we turn to for our personal, emotional and spiritual needs.” This increased investment in groups resulted in a quadrupling of Facebook groups in the subsequent 2 years [4]. By 2020, estimates suggested that 1.8 billion Facebook users were group members of approximately 50 to 100 million groups, and half of all Facebook users were members of ≥5 groups [4]. Given the popularity of Facebook, the time users spend on the platform, and the emergence of private groups, Facebook presents a unique opportunity to deliver evidence-based behavioral interventions in a way that reaches a large audience by meeting people where they are.

Many Facebook users already use the platform to discuss health as evidenced by the vast ecosystem of health groups on Facebook. In 2017, Facebook reported to have 6 million health groups containing >70 million members [5]. Health groups on Facebook are typically created by a patient for the purpose of connecting users who have similar health conditions [6-8]. Research on health groups on Facebook reveals they span a wide range of health topics including diabetes [9], cancer [10], hypertension [11], mental health [12], genetic disorders [13], sexually transmitted diseases [14], and long COVID (post–COVID-19 condition) [15], to name a few. Studies of participants of these groups show that they use groups to share personal experiences with a health condition, exchange information about their condition and treatments, and give and receive emotional support [16-20].

Further evidence that patients are using Facebook to discuss and manage their health comes from a study of 2508 US adult Facebook users that found that more than two-thirds (69%) of the users posted about health at least once in the past year and 38% posted about health at least once per month [21]. This appears to exceed the number of people who use mobile health apps: only 20% of men and 17% of women report to be currently using a health app [22]. As such, popular social media platforms such as Facebook may be more conducive to creating a “community of health” than health apps that tend to be narrowly focused on a single health behavior or condition (eg, weight loss) and do not always include social features that allow the formation of groups where patients can coalesce around specific health topics as is the case on Facebook.

## Facebook Groups: A Novel Intervention Modality

Because of the reach and ease of use of Facebook, it is increasingly being used as a modality by which to deliver behavioral and health communication interventions [23-26]. Typically, a Facebook-delivered intervention involves investigators creating a private group and delivering a feed of intervention content for participants to engage with asynchronously. Often a counselor is present and facilitates discussions and provides feedback and support. Any Facebook user can start a group for free and a user who starts a group is referred to as the administrator (ie, “admin”) and this individual may choose to recruit a moderator (ie, “mod”) whose role is to assist with gatekeeping for membership as well as moderating content posted by members. Although Facebook groups can be public or private, for the purposes of developing and testing interventions in the context of research, private groups are recommended to protect the confidentiality of participants and create a confidential space for them to share their experiences and opinions. To be sure, health communication campaigns delivered in the real world occur on public platforms (eg, social media messaging on public accounts, media advertisements, billboards, and community settings), but the use of private Facebook groups to evaluate the efficacy of a health communication campaign or a messaging strategy allows one to examine participants’ reactions to and engagement with messaging in a controlled setting before implementing in real-world public settings.

Private Facebook groups are joined via invitation only, which allows admin control over entry, thereby reducing privacy risks. Members of Facebook groups can post in the group at any time, and all posts to the group by admins or any member appear in the newsfeeds of group members based on Facebook’s content algorithm [27]. Facebook also provides an option for group members to receive notifications when new posts are made, which cues members to visit the group to see new activity. Facebook groups have myriad features designed to assist the admin in running a group and to facilitate meaningful engagement in the group [28]. For example, admins can schedule posts in advance, which is a useful feature for behavioral interventions because they typically involve a collection of posts (ie, content library) that are posted over an extended period. Posts to the group can include text, images, videos, documents, or polls. Admins can also create a repository of document files in the group for members to access at any time. This is a useful feature for investigators who want to distribute handouts, worksheets, or other resources to participants. Facebook groups also allow admins to create chats on specific topics with subsets of group members and host live events such as question and answer sessions, webinars, or meetings within the private group interface. This allows investigators to incorporate synchronous intervention content directly within the Facebook group, precluding the need to leverage other platforms or videoconferencing technologies. To reinforce member engagement, Facebook provides an array of badges for group members. Group members can earn badges for being a new member, a frequent conversation starter, a founding member, a frequent contributor to conversation threads, and a “visual storyteller” by frequently posting images or videos. Badges appear next to the user’s name wherever they post or comment. Finally, group admins have access to Facebook Group Insights, which allows them to monitor group activity, including post engagement, a list of the most active members, and popular times for posting. Investigators can leverage these features in creative ways to engage members in behavioral strategies and encourage them to engage with and support each other.

## Literature on Facebook-Delivered Interventions

### Overview

**Figure 1:**
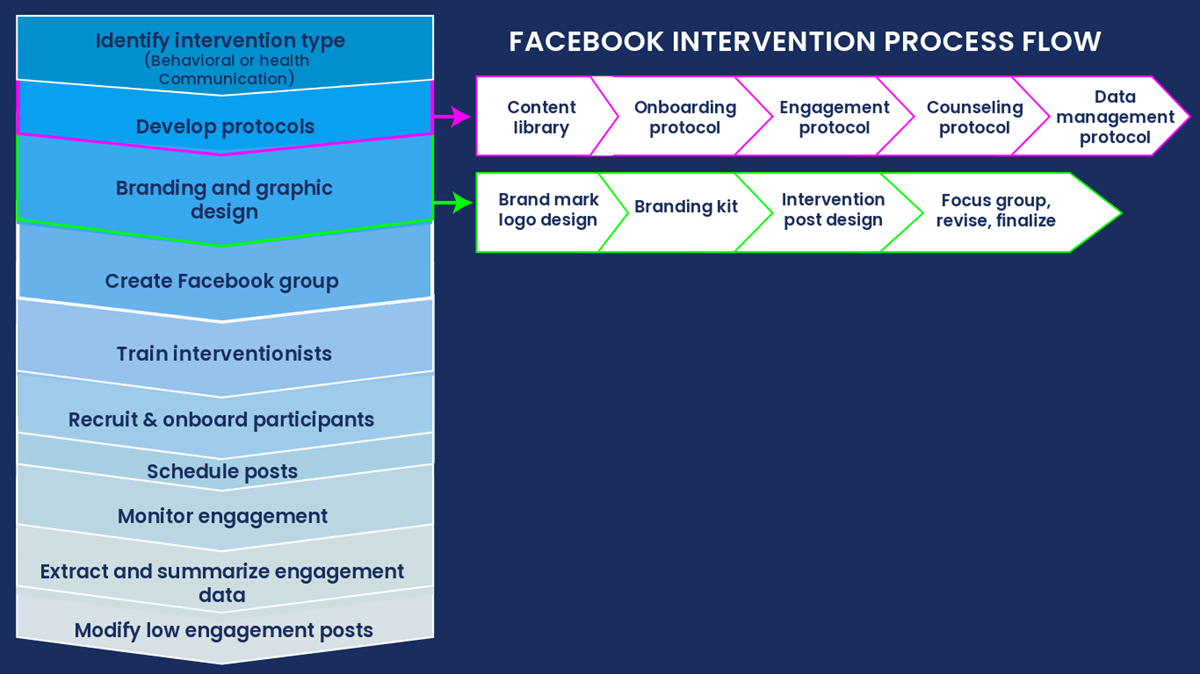
Process of developing a Facebook-delivered intervention.

Studies of Facebook-delivered interventions have been conducted for weight loss [29,30], healthy diet [26], physical activity [25,31], maternal care [32], caregiver mental health [33], postpartum depression [33], preventing cancer recurrence [34], vaccine hesitancy [35,36], smoking cessation [37], and HIV prevention [38], among other topics. Results vary widely in terms of clinical outcomes as well as participant engagement. Engagement tends to be a predictor of outcomes [39-42], but best practices for engaging participants in Facebook groups are lacking [43]. Best practices for adapting and designing behavioral intervention content for asynchronous delivery in Facebook groups are also lacking. We previously described a process for adapting existing behavioral interventions for delivery via social media broadly, with guidance on platform selection, inclusion criteria, content creation, interventionist training, and data reporting [44]. We now build on that work by proposing best practices specifically for the use of private Facebook groups for the delivery of behavioral and health communication interventions, including converting traditional intervention content into Facebook posts; creating onboarding, counseling, engagement, and data management protocols; designing and branding intervention content; and using data to optimize engagement and outcomes (Figure 1). Textbox 1 provides a glossary of terms.

**Glossary of Facebook group intervention terms.**

**Keywords and definitions**

- Group moderator
  Person who reviews all group member posts and comments to ensure they meet community standards.
- Group administrator (admin)
  Person who started the group and establishes the community standards. They may also play the role of moderator.
- Content library
  The entire collection of intervention posts.
- Discussion thread
  A Facebook post that starts a discussion and the collection of replies made by group members in response to the post.
- Poll
  A Facebook post that allows group members to answer a multiple choice question or vote from a list of choices.
- Call to action
  The segment of a Facebook post that asks participants to respond in a certain way (eg, answer a question, share an opinion).
- Brand identity
  The visual elements (eg, name, logo, images, fonts, color palette) of intervention content that create a recognizable identity for the intervention posts so that group members can easily identify intervention posts in their feed.
- Brand kit
  The collection of fonts, color palettes, and graphic assets that are used to express the brand identity in intervention posts.
- Microcounseling or asynchronous counseling
  A form of counseling that occurs in Facebook groups in which counselors engage with group members asynchronously in discussion threads.
- Engagement
  Any visible evidence of participation by group members including reactions (eg, “likes”), comments, replies to comments, poll votes, or original posts on the group wall.
- Engagement protocol
  A protocol that outlines steps for re-engaging group members who have not engaged in a specified period.
- Tagging
  Tagging is a written mention of a group member’s name which then results in that member receiving a notification that they have been mentioned by someone in the group. Clicking on the notification will lead them to the post in which they were mentioned.

### Identifying Your Intervention Type

#### Overview

Facebook groups are not an intervention in themselves; rather, they serve as a platform through which interventions can be delivered. A group with no content is simply a web-based gathering space, which in itself is unlikely to have an intervention effect on any behavioral or clinical outcomes. The first step in developing a Facebook-delivered intervention is to identify the type of intervention to be delivered. The 2 types of interventions that can be delivered are behavioral interventions and health communication interventions.

#### Behavioral Interventions

A behavioral intervention is a collection of behavioral strategies that teach people essential skills for developing adaptive behaviors or discontinuing maladaptive behaviors with the ultimate goal of impacting a clinical outcome [45]. Behavioral interventions often have intensive protocols and traditionally have involved multiple visits with a behavioral provider. Examples include the Diabetes Prevention Program (DPP) Lifestyle Intervention [46], behavioral activation for depression [47], and cognitive behavioral therapy for insomnia [48]. Some behavioral interventions require licensed mental health professionals to deliver (ie, treatments for mental disorders) while others (eg, lifestyle interventions) can be delivered by trained paraprofessionals (eg, health coaches). Behavioral interventions provide intensive support because they target health issues that require the adoption of many new habits. For example, lifestyle interventions target myriad habits relating to diet and physical activity, as well as habits relating to time management, problem-solving, goal setting, and planning [49]. Behavioral interventions are typically administered to individuals or small groups in person or via telehealth, SMS text messaging, mobile apps, and web-based platforms, and they can last weeks or months and even up to a year or more. They are lengthy in duration because participants need time to learn and practice new skills, set goals, troubleshoot obstacles, receive feedback and guidance, and build consistency and support to adopt and maintain the new habits they are developing long-term. Although behavioral interventions are long in duration, relapse and backsliding are common when interventions are terminated, making long-term maintenance of behavior change an elusive goal [50,51]. A promising feature of Facebook groups is that once built and a critical mass of engaged members coalesces, they have the potential to be self-sustaining for years, which may help people tackle issues that arise over time and compromise long-term maintenance.

#### Health Communication Interventions

Behavioral interventions can be contrasted with health communication interventions that use repeated messaging to prompt an audience to engage in a healthy behavior, such as vaccination, mask wearing, or cancer screenings. Health communication interventions also impact clinical outcomes and are often focused on disease prevention. Health communication interventions tend to involve repeated health messaging that is designed to increase a target population’s motivation to take a health action [52] (eg, vaccine) by increasing the perceived risk of the disease and perceived benefits of the health behavior, providing reminders, providing countermessaging to combat misconceptions, and providing resources that eliminate barriers (eg, a link to make a vaccine appointment at a local pharmacy), among other strategies. They may also be used to connect people to behavioral interventions. For example, the Truth Initiative’s *This is Quitting* campaign disseminates videos and print materials (the health communication intervention) to connect youth who use e-cigarettes to a text-based e-cigarette cessation intervention (the behavioral intervention) [53]. Health communication campaigns can occur over lengthy periods as well and often leverage print materials, billboards, social media, media, or opinion leaders and influencers to disseminate messaging to a large audience. A Facebook group for a health communication intervention is a vehicle to disseminate repeated messaging on a topic and may be used to test different messaging strategies (eg, gain- vs loss-framed messages).

The distinction between behavioral interventions and health communication interventions is important from an intervention development perspective. Behavioral interventions require complex skill building and support to change habits; thus, people who enroll in them are typically seeking help for the target condition, and as such, have some level of motivation to change. For example, people who enroll in weight loss interventions do so because they want to lose weight. However, health communication interventions are designed to reach people who are not necessarily actively seeking help, including people who may not believe the health behavior is even relevant to them or healthy at all (eg, vaccine skeptics). For example, influenza vaccine campaigns are designed to reach people who have insufficient motivation to get the influenza vaccine. This motivational distinction is important because people who are not motivated to engage in a healthy behavior are unlikely to join a Facebook group on that topic; however, people who are motivated to change but need help to do so will be far more likely to join a Facebook group on that topic. As such, for the health communication intervention, the Facebook group topic should be one that is sufficiently engaging to the target audience to motivate them to enroll. For example, a Facebook-delivered health communication intervention aimed to decrease mothers’ willingness to allow their teen daughters to use tanning beds via a Facebook group that was themed on the broader topic of teen health [54]. Only 15% of intervention posts were relevant to tanning beds, whereas the remainder of the intervention posts covered a host of health topics that moms rated as high interest in pretrial focus groups [55]. Once the investigator determines whether the intervention is behavioral or health communication, the next step is to develop the intervention protocol that includes 6 components: a content library, branding and graphic design, an onboarding protocol, a counseling protocol, an engagement protocol, and a data management protocol. Interventionists using attention-control Facebook groups in their trials will need to develop similar protocols for their attention-control group as has been done elsewhere [54,56-58].

### Content Library for Behavioral Interventions

Interventionists may want to develop a Facebook-delivered version of a behavioral intervention that has established efficacy when delivered via other modalities such as in-person visits or telephone; alternatively, they may want to develop a new intervention that is to be delivered for the first time via Facebook groups. A review of the intervention literature is a necessary first step to determine if evidence-based interventions for the target health issue exist, and if so, building off of this literature is recommended. For example, if an investigator is interested in helping a specific population segment with depression, a review of the literature will reveal that many depression treatments have been tested using a variety of modalities [59]. The first step then would be to use protocols for existing interventions as a starting point and if cultural or other tailoring is necessary, doing the proper developmental work [60] to finalize the intervention content so that it can be converted into a format for Facebook delivery. If an intervention has already been delivered via a Facebook group in other studies, as in the case of lifestyle interventions [42,61-63], the investigator should then design an intervention protocol that improves upon weaknesses identified in previous studies. If no behavioral interventions exist for the target health issue, the interventionist should take the proper steps to conduct behavioral intervention development, which have been described elsewhere [45]. If the investigator does not have expertise in behavioral intervention development, partnering with a content expert is highly recommended for a first attempt at developing a behavioral intervention. A proper behavioral intervention protocol includes weekly modules that contain learning objectives and content to be covered for each week of the intervention (eg, behavioral strategies, discussion topics, and homework), as these will be the foundation by which to produce a content library of Facebook posts. Traditional behavioral interventions have intervention protocols, but when developing a new intervention, drafting an intervention protocol is recommended before attempting to create Facebook posts because it should be used to guide the development of intervention posts [64,65].

### Converting Intervention Content Into Facebook Posts

**Figure 2:**
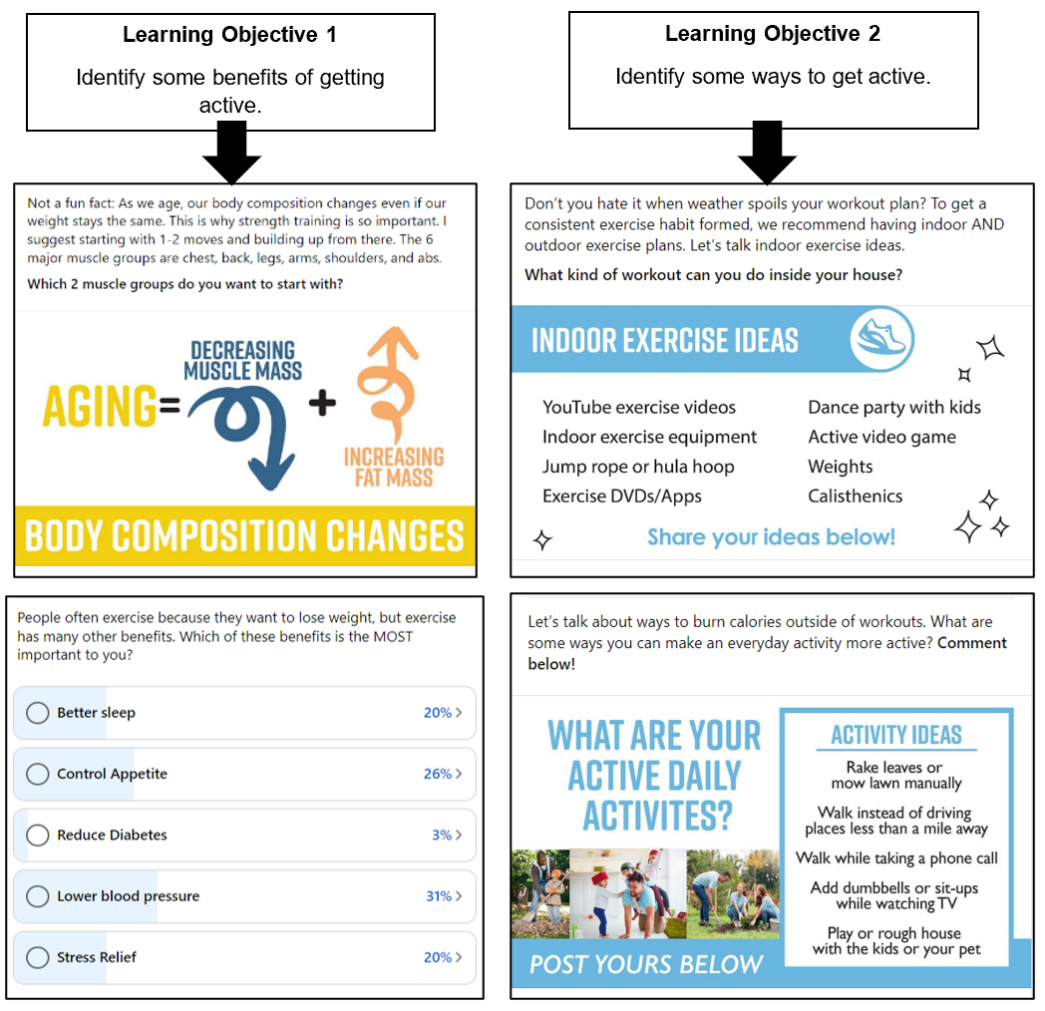
Converting learning objectives into Facebook posts.

We will use the 16-module DPP Lifestyle Intervention [49] as an example for converting a behavioral intervention into Facebook posts. Each module of the DPP has learning objectives and facilitator and participant materials meant to be delivered in a 60- to 90-minute session. With this foundation, the intervention protocol can be converted into any number of modalities including a Facebook group. We converted each of the 16 DPP modules into 1 week of posts (14 posts) that are meant to be distributed 2 per day, 1 in the morning and 1 in the evening. This resulted in a library of 224 posts. In Figure 2, we provide examples of how learning objectives were translated into Facebook posts. Once each learning objective is reflected in a Facebook post or posts, we recommend having a second investigator who has experience with the intervention to independently review the posts to verify that the learning objectives are adequately met to ensure that the Facebook posts have high treatment fidelity.

Like many behavioral interventions, each DPP module includes recurring behavioral strategies (ie, weigh-ins, goal-setting, check-ins on the previous week’s goals, and problem-solving). We designed 4 posts that occur at the same time every week for these recurring behavioral strategies so that participants are prompted to engage in them every week. One benefit of asynchronously delivered interventions is that the interventionist can select optimal days for posts to occur depending on the nature of the post. We selected specific days and times for the recurring posts to appear each week to allow participants to engage in these strategies at times of the week when they would benefit the most. For example, the goal setting post appears every Monday morning so that participants receive diet and physical activity goals at the beginning of the week and have the entire week to work on them. The post that checks in on how participants did on their weekly goals appears each Sunday evening, the last day before they receive the following week’s goal. This allows participants to share how they did with their goals, obstacles that got in the way, and a plan for overcoming those obstacles in the following week to prepare them for a more successful week. The “weigh-in” post appears each Friday morning (the last weekday of the week) so that participants are cognizant of their weight before the weekend begins that may offset weekend overconsumption, which is common [66]. The problem-solving post appears on Wednesday mornings, which is a point at which participants have had a couple of days to work on their weekly goals and may be encountering obstacles that if solved before the week is over could set them up for a successful week. The regularity of these posts provides participant with a predictable structure for the intervention, which is important when intervention content is delivered asynchronously and in such small segments relative to traditional formats where the entire module would be delivered via a 60- to 90-minute synchronous discussion. The remaining 10 posts of the week are designed to tackle the learning objectives of each module while leveraging content in the protocol for that module. The theme of the week is announced in the first post of the week to make participants aware of the discussion topic for that week.

### Content Library for Health Communication Interventions

Health communication interventions typically involve a set of messages that are developed based on a conceptual model and meant to be delivered repeatedly over some period. For example, a health communication intervention based on Prospect Theory might set out to compare gain- versus loss-framed messages about a health behavior [67]. In one study, this was accomplished by randomizing participants to Facebook groups that used gain- or loss-framed messages to improve physical activity motivation [68]. Alternatively, a health communication intervention based on Transportation Theory might set out to evaluate the effectiveness of narrative-based messaging about a health behavior [69]. Social marketing principles, which address how to influence a target audience by developing messaging that resonates with their values, can also provide a guiding framework [70]. Specifically, this framework outlines steps, including identifying a target audience and its unique barriers to the target behavior, gathering data on the target audience’s values, developing messaging that resonates with the audience’s values, and disseminating messaging using channels used by a high proportion of the audience. These are just a few of the many conceptual models that have been used in health communication interventions [71]. Once an investigator has identified a conceptual model, the next step is to begin drafting a library of messages that reflect the appropriate theoretical constructs. As recommended earlier, the entire feed in a health communication intervention should not be exclusively focused on the target health behavior because people who are not motivated to learn about that behavior may be unlikely to join. For example, a Facebook group on vaccines would not attract the interest of people who are vaccine hesitant, in which case 2 problems are likely to occur. First, recruitment may be slow and difficult to accomplish. Second, the recruited sample is unlikely to be representative of the target population because people who volunteer to participate in a group solely focused on vaccines will naturally have more interest in vaccines. This increases the risk of ceiling effects that reduce the power to detect an intervention effect. Preliminary work that queries a representative sample of the target population on topics of interest is an approach that has been successful in previous work [54,55]. A broad topic that encompasses the target behavior will allow messaging about the target behavior to fit into the content feed while also not overpowering it. Once a topic is identified, the investigator will need to develop messaging on that topic. In our previous studies, 15% of messages were on the target behavior and 85% of messages were on the broader topic and this was sufficient to produce an intervention effect [36,54]. The investigators may or may not use theoretically-based messaging strategies in posts relating to the broad topic but doing so gives those messages scientific value, makes the feed consistent, and may provide interesting data for secondary analyses.

## Best Practices for Developing Facebook Posts for the Content Library

### Text

The typical intervention post includes text and some form of media (eg, image, video, and link) or polls. While Facebook has a generous character limit of 63,206 characters for posts, social media marketing experts recommend keeping the text at approximately 40 to 70 characters because shorter posts tend to get more views by being easier to read and comprehend when users are scrolling, especially on a mobile device [72]. Posts that exceed 2 lines of text will require a user to click to “read more” if they are viewing on a mobile device and those that exceed 5 lines of text will require a user to click to “read more” if they are viewing it on a computer. Pithy posts that do not exceed these limits are ideal because if the initial text does not draw the user’s interest enough for them to click “read more,” the opportunity to engage them in the intervention content captured in that post will be missed.

Another important consideration in drafting the text of an intervention post is how to engage participants with the intervention content in that post *and* the group itself. Human-delivered behavioral interventions are interactive, which entails exchanges not only between counselor and group members but also between group members. Group cohesion is an essential ingredient in group-delivered interventions and refers to the sense of belonging, interpersonal support, and acceptance that is generated from group interactions [73]. In the group therapy literature, group cohesion is associated with better attendance, greater interpersonal support, and better outcomes [73]. The ideal intervention posts will engage group members in a behavioral strategy and facilitate group cohesion. In Facebook-delivered interventions, the investigator should design posts that start conversations in ways that closely emulate how the intervention would be delivered offline. Posts should have a “call to action” such that participants are asked to share something in the comments, thus creating a “discussion thread” on a topic. The call to action might involve a question (eg, “what is the hardest part about exercising on the weekends?”), brainstorming (eg, “what are some ways to avoid nighttime snacking?”), soliciting experiences (eg, “how did you do on the fruit and vegetable goal this week?”), or soliciting opinions (eg, “what is your favorite healthy dessert?”). A major challenge in conducting asynchronous web-based groups is that they lack some interpersonal connection-building aspects of an in-person group such as nonverbal behavior (eg, eye contact), the ability to have a synchronous dialogue, and the ability to connect one’s story with their emotional and physical characteristics including their voice, facial expressions, and body language. For example, in an in-person group, a group member might tell a very moving story about overcoming a difficult challenge, and her facial expression, tear-filled eyes, and cracks in her voice are likely to stimulate emotional reactions and empathy among fellow group members. However, the same story typed into a comment on the internet without the nonverbal interpersonal experience may not generate as intense of an emotional reaction among group members, especially if the comment is viewed by a group member as they are casually scrolling through their Facebook feed without full attention to the content they are viewing. As such, providing as many opportunities as possible to engage group members, pull them into discussions, stimulate emotional connections, and cue them to share their experiences are all essential to building group cohesion in this format. Table 1 provides examples of conversation starters.

**Table 1:** Examples of conversation starters and targeted behavioral strategies.

Conversation starters and stems		Posts	Behavioral strategy
**Open-ended questions**			
	What...	Time to weigh in! What was your weight change this week?	Self-monitoring
	How...	How will you plan on being active this weekend?	Planning
	When...	When did you find your motivation to exercise start to slide?	Problem solving
	Which...	Foods rich in fiber can help to control appetite. Which high-fiber food on this list could you easily eat more of?	Goal setting
**Open-ended statements**			
	Share...	Take a look at your MFP diary from the past week and identify ONE food you could replace with a lower calorie option that will save at least 100 cals. Share your swap in a comment!	Goal setting
	Name...	Building muscle can help you break through a plateau because muscle burns calories even at rest. Name a strength exercise you do regularly and which muscle groups it works!	Positive reinforcement
	Reply with...	Surrounding yourself with things that inspire you to eat healthy is just as important as eliminating temptations. Reply with ideas for things that inspire you to eat healthy.	Stimulus control
	Add yours...	Who has a sweet tooth after dinner? Let’s brainstorm some healthy, low calorie dessert ideas. Add yours in a reply. I’ll list a bunch too!	Problem solving

### Images

The image in a post is most often the first thing a Facebook user will see, even before reading the text. The image heavily influences the user’s decision to read the text. As such, images are an opportunity to draw group members into the post. Posts with images also tend to elicit more engagement [74], therefore it is highly recommended to include an image in a text-based post. The image should resonate with the message of the text such that once the group member is drawn in, they will find that the text complements the ideas and themes reflected in the image. Using an image that is attention grabbing but unrelated to the message of the text may confuse participants or result in their feeling baited into a discussion they were not expecting, which may discourage engagement not only on that post but also on future posts. Images can contain text, infographics, or pictures. If text is in the image file, it can include the call to action, but then the text of the post would not need to include a call to action as well. Having one call to action per post makes it easier for participants to understand what is being asked of them and how to respond. For example, if the post asks participants to both set a goal and do problem-solving, the discussion thread might include some participants doing one or the other or both, which means the discussion thread will have different discussions going on within it, rather than everyone focused on sharing the same thing and reacting to what each other has shared. Another consideration when posting images is ensuring they appear in high quality when posted, that the text is large enough to read on mobile devices, and that the images are of the optimal size for posting on Facebook. Creating a “test group” to review posts on both mobile and desktop before they are used in an intervention can help to identify edits that should be made to improve clarity and readability.

### Videos

Videos can be an excellent tool for conveying complex information that cannot be captured in a text-based post and for the group to become better acquainted with the interventionist. A brief introductory video shared on the first day of the intervention where the interventionist introduces herself and gives a brief overview of the program can be useful in building a relationship with the group. The frequency of videos is at the interventionist’s discretion, but videos require participants to click on them to receive the content, which may result in fewer participants viewing that content. To increase the click rate, the text for the video and the video thumbnail should pique participants’ interest. The maximum video length allowed on Facebook is 240 minutes, however, this length of video would not likely be viewed by many participants. Facebook recommends keeping videos to 15 seconds to maximize the chances the viewer will watch them to the end [75]. The first 3 seconds of the video should be designed to draw the participant’s interest enough for them to continue watching and is the ideal place to insert the most important part of the message. Including captions will also increase the likelihood that participants will view the video because captions allow participants to digest the content without audio, which is inclusive of people of all abilities and useful for a participant who is in a location where they cannot play audio. Videos do not need to be professionally produced as “home grown” videos shot on mobile phones are extremely common on social media and they are inexpensive to create. The use of teleprompter mobile apps while shooting a video can help the subject of the video appear more natural when communicating into the camera.

### Polls

Polls tend to attract more engagement than other posts [39], which presents a unique opportunity to use them to solicit experiences and opinions from participants that they might not otherwise share via other post types. Polls can also be used to help participants with goal setting, testing their knowledge, increasing accountability, and problem-solving (Table 2 describes poll discussion starters). For example, polls may be used for problem-solving by designing them to solicit barriers to a behavior change. In our lifestyle intervention, we use weekly polls to solicit barriers to diet and physical activity. Each poll option will show the names of participants who selected it, which allows the interventionist to follow up in the comments and engage in problem-solving with participants who selected each option. Polls should be used as discussion starters because they may not contribute to an intervention effect if the participant simply selects an option and no further discussion ensues. Having the interventionist address what they learned about participants in the poll by creating comment threads that tag participants who selected different options is a way to use polls to engage participants in a behavioral strategy.

**Table 2:** Examples of polls and targeted behavioral strategies.

Poll	Response options	Behavioral strategies
An important skill in developing new habits is planning. When it comes to exercise, how far in advance do you plan a workout?	I don’t plan workouts at all I plan my workout on the same day I plan my workout the day before I plan my workouts for the week at the beginning of the week	Planning
When is the best time to drink water to enhance weight loss?	First thing in the morning All day long Immediately before a meal Right after a meal	Assess knowledge
Special events are fun but can be the source of frequent setbacks. Today’s question is: Which of these almost always causes you to gain a pound or two?	Holiday weekend Vacation Work travel Birthday Thanksgiving All of the above	Problem solving
Strength training is essential to preventing muscle loss with aging. Which muscle group are you most motivated to start with?	Arms Back Abdominal Shoulders Legs	Goal setting

### Links

Facebook posts can also include links to other content such as articles, websites, and other resources. A drawback to using links to deliver intervention content is that if the substance of the post is behind the link, only participants who click the link will receive it. If it is possible to simply put the key message directly into the text of the Facebook post, the proportion of participants who receive it will be higher. As such, links are best used as a resource for additional information or reading beyond what is included in the post rather than for the delivery of essential intervention content. Another drawback of links is that they drive participants out of the Facebook group and to another website and this may reduce the likelihood they will engage on the content in the group. For example, a participant may click on a link to an interesting article and then proceed to engage in the comment section of that article or continue browsing that website rather than the group. We recommend using links sparingly and instead creating a resource library that participants can access at any time. In our lifestyle intervention, we created a Pinterest page that includes links to recipes, meal plans, workout videos, and other resources for participants to use as a resource library [76]. This prevents informational content from taking up too much space in the Facebook group because such content is not likely to start conversations. When participants ask for resources, the counselor can then reply with a link to the Pinterest page.

## Branding and Graphic Design

### Purpose

Branding and graphic design are essential elements in the building of behavioral interventions delivered through Facebook groups. These disciplines are not merely for esthetic considerations, they are instrumental to effective communication, comprehension, reinforcing the core values and messaging in an intervention, and enhancing engagement, and ultimately, the impact of the intervention. Graphic design involves leveraging visual elements and design principles to communicate ideas and concepts [77]. A brand is “any distinctive feature like a name, term, design, or symbol that identifies goods or services,” according to the American Marketing Association [78]. For example, yellow arches with a red background are highly recognizable around the world to be the McDonald’s brand. Brand identity is the mix of visual elements, including the program’s name, logo, symbols, typography, and color palettes that people recognize and associate with the brand. Brand identity can also be conveyed in other elements of the program, including recruitment advertisements and the program website. Just as commercial marketing uses branding to drive behavior change for revenue generation, branding can be useful to drive behavior change when designing content for Facebook-delivered interventions. Branding and graphic design help establish the identity and credibility of an intervention, and as a result, may enhance participant engagement by drawing participants’ attention to intervention content. Adding a graphic designer to your team is a best practice as their expertise ensures that the visual representation of the intervention is not only appealing but also strategically aligned with the conceptual model and behavior change strategies being used. This synergy is key in crafting a distinct presence in the digital realm, where recognizable elements such as a program’s name, logo, and design need to be consistent and impactful to capture the fleeting attention of users scrolling through their web-based newsfeeds. The process of bringing a program’s brand identity from concept to community requires that each visual element aligns with the overarching goals of the intervention. In terms of budgeting, in addition to the graphic designer’s fees, budgets should include licenses for collaborative tools such as Canva (Canva Inc) or Adobe Express (Adobe), which help streamline the design process and enable development, sharing, editing, reviewing, and storage of intervention content. If the project budget is lean, investigators can explore collaborations with fine arts or graphic design departments at their university to identify students who have proficiency in using design tools.

### Organizing Branding and Graphic Design: Key Elements

The journey from conceptualizing a program’s brand identity to the final implementation of its content in a Facebook group requires continual collaboration between a graphic designer and investigator. Incorporating a graphic designer from the onset ensures that branding is not an afterthought but a foundational component of program development. Before beginning, take time to budget appropriately and establish timelines and regular communication channels, such as weekly meetings, to ensure progress and alignment with the intervention goals. The brand identity and design process involves these elements:

- Program name: determining the program name is the initial step in creating a brand identity. The name should be pithy, capture the essence of the program, and be memorable to the target audience. Jargon should be avoided, including the use of the term “intervention” given it conjures different meanings when used colloquially.
- Audience defined: understanding the audience is crucial to designing a brand that resonates with that audience. The demographic, psychosocial, and behavioral characteristics of the target audience should guide the visual and communication strategies of the brand.
- Logo design: the logo, also referred to as a brand mark, is the visual cornerstone of the brand identity. An effective logo should be distinctive and relevant to the health program’s mission. It should also be visually effective across various formats, including web, mobile, and branded promotional items (eg, mugs and magnets). Some universities require alignment with the university brand kit when creating logos and branded materials. Identify and communicate what standards your program and designer need to follow.
- Brand kit developed: a comprehensive brand kit includes fonts, color palettes, and graphic assets. These elements ensure consistency across all materials and platforms. A brand kit can also include templates to aid in future intervention content development.
- Finalize intervention content conversion: content conversion involves adapting the intervention materials into individual posts, including copy, a visual idea, a call to action, and a post type suitable for Facebook group nuanced delivery. The text content of each post should be finalized before branding and design.
- Decide on post type balance: a balance of post types (polls, images, videos, etc) is necessary to maintain user engagement and cater to different content consumption preferences. Overuse of any one type of post could result in participant fatigue for that post type. For example, although polls tend to get high engagement, the use of several polls in a week could result in the feed becoming monotonous, which could result in participants disengaging.
- Graphic designer designs posts: after receiving the converted intervention content, the graphic designer uses the brand kit to design the posts in a way that aligns with the brand identity.
- View posts on all device types: once posts are designed, they should be viewed on both desktop and mobile platforms to evaluate readability, compatibility, and accessibility. Posts that are difficult to read on any platform should be modified. This ensures that the content’s integrity is maintained across all platforms participants might use to view it.
- Focus group testing of posts: focus groups conducted before and after pilot studies can be used to evaluate posts in terms of clarity, comprehension, valence, and persuasiveness. Focus group participants can also share how likely they would be to engage with the post and how they would engage, which reveals whether the post is likely to elicit the type of engagement intended.
- Post revision: posts should be revised based on the feedback from testing and may require multiple iterative rounds of testing and revision.
- Content library and posting: software programs such as Canva or Adobe Express can be used to store and edit the entire collection of posts (ie, content library). Posts can then be scheduled for Facebook group posting directly from the content library or via Facebook.
- Data-driven modifications: once the intervention ends, post engagement data should be inspected to identify posts that are in the bottom quartile of engagement. These posts can then be compared with those in the top quartile of engagement to glean possible reasons for low engagement. This will help guide post modifications to improve engagement in the future.

### Collaborating With Graphic Designers

Graphic designers are unlikely to have deep knowledge of the intervention topic, thus intervention content development must occur via close collaboration between the investigator (content expert) and the graphic designer. The investigator is responsible for ensuring that the conceptual model, behavioral strategies, and intervention learning objectives are expressed in posts, while the graphic designer is responsible for ensuring the visual elements align with those features and resonate with the target audience. The graphic designer should be included in team meetings to ensure they are knowledgeable about not only the intervention but the study itself.

## Onboarding Protocol

Because Facebook-delivered interventions are a unique experience relative to user-initiated Facebook groups, an onboarding protocol that helps participants know what to expect and how to engage can prepare them to be actively engaged participants and get the most out of the group. Onboarding can be done via a telephone call, webinar, or written materials. It is important to choose a modality that allows the research team to assess the participants’ understanding of the intervention and gives participants the opportunity to ask questions. We conduct 1-hour onboarding webinars before randomization to discuss Facebook privacy policies related to groups, the origin of the intervention, the intervention goals, the recurring weekly posts and how to respond to them, how to participate in the group including how to post and the option to post anonymously, how to earn engagement badges, and how we will extract data from the group and what we do with that data. During the webinar, participants are given the opportunity to ask questions and are also asked to share any anticipated barriers to participation. The use of onboarding webinars such as this has been shown to improve retention [79].

## Counseling Protocol

**Figure 3:**
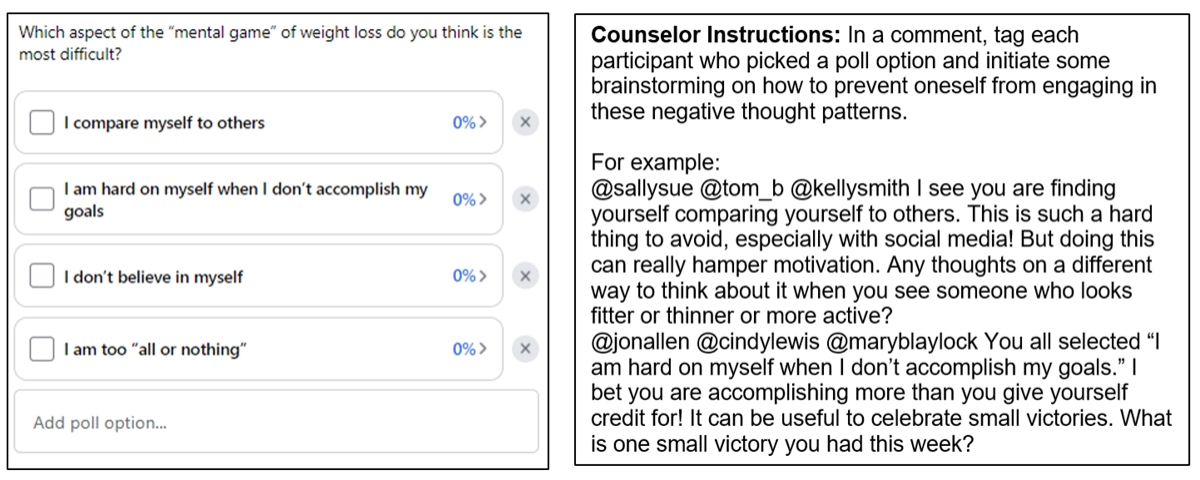
Poll with counselor instructions.

The counseling protocol provides guidance for counselors about their role in an asynchronous web-based setting. Asynchronous web-based counseling, which we have referred to as “microcounseling” elsewhere [44], is different than in-person or synchronous counseling in that a written post starts the conversation and some group members respond and usually at different times, and other group members do not respond at all. If the counselor is too passive, participants who are initially engaged may exhibit declining engagement when they see they do not get meaningful responses, and participants who do not engage much initially may never engage very much throughout the intervention. A proactive counselor can be instrumental in setting the tone for a highly engaged group and is an important role model for engagement in the group. The counseling protocol includes a basic orientation to Facebook groups and their features; an orientation to the content library; instructions for how to engage on each post (Figure 3); how to record brief videos; and scripts for brief videos, live chats, or live group meetings. A detailed protocol will be useful, particularly for counselors who do not have experience in this setting. Counselors with no experience in this setting should be trained in advance and shadow a more experienced counselor until they feel comfortable leading a group alone. Regular counselor supervision meetings can be useful to help them navigate challenging participant situations including disengaged participants.

## Engagement Protocol

As in any behavioral intervention, some participants disengage over time and this puts them at risk for dropout, and ultimately, treatment failure. Numerous studies show that engagement in Facebook-delivered interventions is a predictor of treatment outcomes [41,80], so having a protocol to maximize participant engagement at the outset and to reengage participants who exhibit declining engagement may enhance retention and outcomes. Engagement is a key variable in the algorithm Facebook uses to determine the order in which content appears in an individual user’s newsfeed [27]. Facebook’s algorithm is proprietary, but generally it “scores” content by how much it predicts the user will enjoy that content, and that score is based on the user’s past engagement with content from that source and how popular the content is among other users. It then arranges the user’s feed in such a way that the content that is prioritized is the content the algorithm predicts the user is most likely to enjoy [81]. Because the degree of engagement in a Facebook group overall is a factor in the algorithm, it is important that intervention content attracts participant engagement. A poorly engaged group may result in declining post views (and thus engagement) by all group members simply because content from the group will not be prioritized by the algorithm [81].

The objective of an engagement protocol is to provide a structured plan to maximize participant engagement. During onboarding, participants should be instructed to add the group to their “Favorites” list because Facebook’s algorithm prioritizes content that users put on their “Favorites” list [81]. Participants can accomplish this by clicking on their account icon, going to Settings and Privacy, selecting Feed, and then choosing Favorites. They can then find the group on the list of items they follow and add it to their Favorites. Users can add up to 30 items to Favorites.

During the intervention, investigators should track engagement data weekly to identify participants who are becoming disengaged. Facebook group insights can be used to identify participants who have not engaged in the past week. which is a point at which reengagement attempts may be indicated. Once disengaged participants are identified, the counselor can attempt to reengage them in a few ways. First, they can “tag” a participant in a reply to a post, asking them for their thoughts on the post. This simply entails typing the participants’ name into the post, which then sends them a notification that they were tagged; however, if the participant turns off notifications from the group, they will not be notified that they were tagged in a post. Limiting the number of participants tagged in one comment may be prudent because tagging many participants in a single comment on a post could result in a diffusion of responsibility to reply and therefore, be counterproductive to increasing engagement. Tagging should be used sparingly and judiciously to avoid a participant feeling singled out after being tagged repeatedly. If a participant does not respond to being tagged, the counselor can send a private Facebook message to the participant to attempt to reengage them. If the participant is not logging into Facebook often, they may not receive private messages in which case the counselor could send an SMS text message, email, or call them on the phone.

Another problem that can be addressed in the engagement plan is what to do when specific intervention posts garner little or no engagement. If a particular post is not getting much engagement in the first 12 hours of posting, the counselor can tag the entire group in a comment by starting the reply with “@everyone” which will send a notification to all group members that they have been tagged in a comment. Once the intervention ends, the investigator should sum engagement data (eg, likes and comments) for each post and identify posts that are in the bottom quartile for engagement. Comparing these posts to those in the top quartile may elucidate characteristics of posts that elicited very little engagement and can be useful to guide intervention content refinements. For example, low-engagement posts might have a missing, cryptic, or confusing call to action, or the call to action asked group members to share something many people might not be comfortable sharing (eg, “Share a time when you ate far more than you planned”). Other factors to look for in low-engagement posts include high character counts, long duration videos, use of links that may have driven participants away from the group, or images with hard-to-read fonts. Another reason for low engagement might be that the content of the post was not inclusive to all group members. For example, the post may have only resonated with group members of certain racial and ethnic backgrounds, sexual orientations, body sizes, genders, ages, skin tones, or life circumstances (eg, marital status). Investigators can also inspect the inclusivity of discussion threads for each post by examining who participated in the thread and who did not. Postintervention focus groups that query participants on how inclusive the feed felt to them can provide valuable information for refining intervention posts.

Some investigators, particularly those who are using a set of intervention content for the first time, might discover that a high proportion of posts received very little engagement. This may occur for a variety of reasons. One reason may be that many participants in the sample are not active on Facebook. In addition to low engagement, another sign that participants are not active on Facebook is low view counts on intervention posts. For each post, Facebook shows data on how many group members viewed the post. If only a small fraction of participants viewed a large proportion of intervention posts, this raises the possibility that the sample may include too many people who are not using Facebook often. This can be remedied in future studies with inclusion criteria that require participants to log into Facebook regularly (ie, several times per week). Facebook-delivered interventions are best matched for regular users of Facebook because they are already in the habit of logging into Facebook and reading their newsfeeds. When recruiting participants who are not regular users, the investigator has the additional task of cuing participants to log in. This can be accomplished by having them set up email notifications when a new post is made in the group or scheduling a daily reminder to visit the group. Low engagement on numerous posts could also be due to the factors relating to post quality as discussed previously. For example, if a large percentage of posts lack a call to action or have very high character counts, this may inadvertently discourage participant engagement as they may lose interest or feel the feed is too cognitively taxing. We recommend that investigators conduct single-arm proof-of-concept or pilot studies of a brief version of the intervention to pretest intervention content, which can flag low engagement posts so they can be revised before conducting a fully powered randomized trial.

## Data Management Protocol

A Facebook-delivered intervention can yield thousands of reactions, comments, and poll votes from participants, which provides a wealth of opportunities to understand how participants interacted with the intervention content, the counselor, and each other. Although Facebook provides some group engagement insights, unfortunately, it does not provide summaries of participant-level engagement data. In January 2024, Meta announced that the Facebook Groups application programming interface will be no longer supported after March 2024 [82]. This means that software tools to extract engagement data from private groups (eg, Grytics) are no longer functional. The process of extracting engagement data from the group and converting it into a data set containing participant-level engagement in a wide data set format (ie, each participant occupies their row, and each variable occupies a single column) is as follows.

First, the investigator should indicate in the consent form that engagement data will be extracted from the group to be sure that participants understand that their engagement in the group is data under study. An important consideration for engagement data extraction is timing because although 75% of engagement on a post occurs within the first 5 to 6 hours of it being posted [83], additional engagement may occur over the next few days as some participants may not visit the group every day. For this reason, data extraction for a given post should not occur until at least a week has passed. If a participant leaves the group prematurely, their engagement will remain, however, their “views” data will disappear on all posts upon their exit. Thus, extracting engagement data before the intervention ends, which is the point when participants are most likely to exit the group, will preserve “views” data.

Engagement data can be manually extracted by a research assistant into a secure web application (eg, REDCap [Research Electronic Data Capture; Vanderbilt University]). First, the unique ID numbers and the content of the following items should be extracted: (1) the Facebook post or poll, (2) comments, and (3) replies to comments. Then, the author ID and name, time stamp, text, attachment (link, image, and video), the ID of the participants who made reactions and the type of reactions, the ID of the participants who viewed the content, and direct URLs to the Facebook posts and comments should be extracted. Trials may have hundreds of posts and thousands of comments and reactions depending on intervention length and number of participants, thus proper budgeting for this labor is important as data extraction can be a time-consuming task. For quality control, a second research assistant should review engagement data from a randomly selected 5% of intervention posts and record and correct errors. If the error rate is >10%, then the research assistant who did the extraction should be retrained and the remainder of their intervention posts should be rereviewed for errors so any can be corrected.

Manually extracted Facebook engagement data should be imported into a statistical software program (eg, SPSS, SAS, Stata, and R) where the investigator should assign week numbers to each post, comment, reply, and reaction based on the time stamp variable. This allows weekly engagement to be computed and for an examination of engagement trends over time. The investigator should also assign variable names and convert the format of the date and time to be compatible with the database program.

The final Facebook engagement data set should be aggregated to the participant level such that each participant ID occupies its row and each engagement variable occupies a single column. Data in this format is ready for analyses. Engagement data are often not normally distributed; thus, distributions should be explored to identify outliers and determine whether parametric or nonparametric analyses are appropriate. Descriptive statistics can be performed on each form of engagement separately (ie, posts, comments, reactions, and poll votes) and for total engagement (ie, the sum of all forms of engagement). Trend analyses and probability tests can be performed to understand trends in participants’ engagement over the course of the intervention and the posts and types of posts with the greatest engagement. Social network analyses can be used to map participants’ interactions with each other and the group leader and study how the frequency of these types of interactions are associated with outcomes [84]. Qualitative content analysis and natural language processing can be performed to understand the nature of the participants’ conversations, and when combined with statistical models and machine learning–based algorithms they allow researchers to identify important contextual predictors of participant engagement [85].

## Real-World Implementation and Facebook-Delivered Interventions

Facebook is an intervention modality that has the potential for reach and scalability, but investigators must chart the implementation path at the earliest stages of development. Although many pilot studies exist across a wide range of topics [36,42,61,62,86-88], few fully powered trials of Facebook-delivered interventions have been conducted [54,63,89,90] and no implementation or dissemination trials exist to our knowledge, thus this area of research is still in its infancy. Implementation of Facebook-delivered interventions in the real world could happen via partnerships with either on the web or offline entities. Developing web-based partnerships requires identifying web-based communities whose subject matter and content are compatible with the goals and values of the intervention. For example, an efficacious lifestyle intervention may have appeal in the ecosystem of diabetes-focused Facebook groups. Furthermore, an efficacious sun safety health communication intervention for parents of small children may be particularly appealing in parent-focused Facebook groups. In terms of partnerships in offline settings, insurers, clinics, and community health settings may be interested in this low-cost alternative to expensive digital platforms. Engaging and partnering with stakeholders in the early stages of intervention development allows their input to shape the intervention, which will increase the likelihood that the intervention finds its way into a real-world setting.

## Conclusions

Facebook is the most popular social media site for adults [91,92], with more monthly users than any other app in the Google Play store or Apple App Store [93], which presents myriad opportunities to research ways it can be used to improve public health. Both behavioral and health communication interventions can be adapted for Facebook group delivery, and given the immense ecosystem of organically formed groups on Facebook, implementation pathways abound. For example, investigators who have established the efficacy of a Facebook group–delivered behavioral intervention for diabetes self-management could partner with administrators of large existing diabetes Facebook groups to conduct an implementation trial in which members are offered access to a sister group where the intervention is delivered. In terms of efficacious health communication interventions, an investigator who established the efficacy of a health communication intervention on childhood vaccination; for example, they could partner with administrators of large Facebook groups or pages for expecting or new parents. Successful intervention delivery via this modality requires knowledge of how a target audience uses Facebook and how to engage them with evidence-based content. Studies are needed to establish best practices for intervention content design in ways that maximize intervention receipt, participant engagement, and outcomes. For Facebook group–delivered interventions to achieve their potential for impact, scalability, and reach, research should be informed by the science of behavioral intervention development, health communication theory, social media marketing research, and implementation science.

## Abbreviations

DPP: Diabetes Prevention Program
REDCap: Research Electronic Data Capture
